# Skeletal Muscle Characterization of Japanese Quail Line Selectively Bred for Lower Body Weight as an Avian Model of Delayed Muscle Growth with Hypoplasia

**DOI:** 10.1371/journal.pone.0095932

**Published:** 2014-04-24

**Authors:** Young Min Choi, Yeunsu Suh, Sangsu Shin, Kichoon Lee

**Affiliations:** Department of Animal Sciences, The Ohio State University, Columbus, Ohio, United States of America; Huazhong Agricultural University, China

## Abstract

This study was designed to extensively characterize the skeletal muscle development in the low weight (LW) quail selected from random bred control (RBC) Japanese quail in order to provide a new avian model of impaired and delayed growth in physically normal animals. The LW line had smaller embryo and body weights than the RBC line in all age groups (*P*<0.05). During 3 to 42 d post-hatch, the LW line exhibited approximately 60% smaller weight of pectoralis major muscle (PM), mainly resulting from lower fiber numbers compared to the RBC line (*P*<0.05). During early post-hatch period when myotubes are still actively forming, the LW line showed impaired PM growth with prolonged expression of Pax7 and lower expression levels of MyoD, Myf-5, and myogenin (*P*<0.05), likely leading to impairment of myogenic differentiation and consequently, reduced muscle fiber formation. Additionally, the LW line had delayed transition of neonatal to adult myosin heavy chain isoform, suggesting delayed muscle maturation. This is further supported by the finding that the LW line continued to grow unlike the RBC line; difference in the percentages of PMW to body weights between both quail lines diminished with increasing age from 42 to 75 d post-hatch. This delayed muscle growth in the LW line is accompanied by higher levels of myogenin expression at 42 d (*P*<0.05), higher percentage of centered nuclei at 42 d (*P*<0.01), and greater rate of increase in fiber size between 42 and 75 d post-hatch (*P*<0.001) compared to the RBC line. Analysis of physiological, morphological, and developmental parameters during muscle development of the LW quail line provided a well-characterized avian model for future identification of the responsible genes and for studying mechanisms of hypoplasia and delayed muscle growth.

## Introduction

Understanding growth and development of skeletal muscle is one of the most important goals in animal production and human medicine [1]. The quail has been used as an animal model for the muscle growth and development because of its relatively rapid generation time [2], easy access to study embryonic muscle development, and conservation of muscle developmental processes with mammals. With these advantages, several lines of quail have been developed by selection for body weight from the random bred control (RBC) Japanese quail for over 40 generations at The Ohio State University [3], [4]. Selection of quail led to establish a heavy weight (HW) quail line that exhibited more than two times greater pectoralis major muscle weight (PMW) than the RBC quail line, which is accompanied by muscle hypertrophy rather than muscle hyperplasia [5]. However, the selected line of quail for the low weight (LW) line has not yet been characterized for muscle growth and development. Previously, several muscular dystrophy animal models with dysfunctional muscles have been extensively characterized [6]–[8]. Decreased muscle mass in physically normal avian species with delayed muscle growth during development, including the LW quail line, is far from well-known. Thus, understanding developmental characteristics of these muscles as an animal model is necessary for the improvement of muscle growth in animals and humans.

Muscle growth and ultimate muscle mass are largely determined by both initial numbers of muscle fibers and growth of size and length of individual muscle fibers during the postnatal period [9]. Thus, both number and size of muscle fibers are correlated with growth rate and muscle mass [9], [10]. On the other hand, selection for body weight and muscle mass in livestock has altered the muscle fiber characteristics [11]. Broiler-type chickens selected by their growth capacity exhibited a greater number and size of muscle fibers [12], and the pectoralis major muscle is more glycolytic and composed of almost entirely larger type IIB fibers compared to layer-type chickens [13], [14]. Whereas, the pectoralis major muscle of the volant species, including quail, is more oxidative and composed of type IIA and IIB fibers due to their flight behavior [15]. The HW quail line exhibited a higher percentage of type IIB fibers compared to the RBC quail line [5].

There are complex interplays of factors and consequent physiological changes that regulate skeletal muscle growth and development. During development, muscles become adapted to perform specialized and diverse functions, which are accompanied by temporal changes in the composition and level of expression of various myosin heavy chain (MHC) isoforms within muscle, as MHC is the major component of the contractile apparatus of muscle fibers [16], [17]. The embryonic and neonatal MHC isoforms are expressed in a temporal and tissue-specific manner during muscle development, and further these developmental MHC isoforms are down-regulated and replaced by adult MHC isoforms after hatch or birth [16], [18]. There are marked differences in the expression and transition of these MHC isoforms between muscle and breed [17], [19]–[21]. Muscles harboring a higher proportion of fast-twitch fibers showed a rapid growth rate [10], and the developmental MHC isoforms were rapidly replaced by the adult MHC isoforms after birth compared to muscles harboring a higher proportion of slow-twitch fibers in mice [17]. In the avian model, the embryonic to adult MHC isoform transition is occurring faster in chicken breeds selected by their growth capacity than chicken breeds selected by their egg production [21].

Like the transition of expression of MHC isoforms considered as a developmental process of muscle, the paired box transcription factor Pax7 is also a well-known proliferation maker, and is necessary for myogenic cell development and satellite cell specification [22]. Pax7 expression is associated with the existence of active satellite cells, and is important in hyperplastic and hypertrophic growth in skeletal muscle [23]. Moreover, MyoD and Myf-5 have been used as markers of proliferation to differentiation events [24]. The basic helix-loop-helix myogenic regulatory factors, including MyoD, Myf-5, and myogenin, play seminal functions that trigger the expression of myofibrillar proteins and permit the assembly of functional muscle fibers [25]. Muscle fiber hyperplasia occurs with high expression of MyoD and Myf-5 proteins [26]. Myogenin is required for committed cells to terminally differentiate into myocytes and mature into muscle fibers, thus associated with muscle fiber hyperplasia and hypertrophy [27]. Therefore, temporal expression of these myogenic markers is related to temporal expression of developmental MHC isoforms within muscle [21]. Generally, comparison of the expression patterns of these markers among animals with different muscle growth and development would provide a reliable indication of affected developmental process.

Therefore, this study was designed to investigate the differences in histochemical characteristics of the pectoralis major muscle, as well as, temporal expression of MHC isoforms and myogenic markers, and their relationship to muscle growth in the LW quail line compared to the RBC quail line. The main goal of the current study is to provide a new avian model with well-characterized muscle growth and development and to further investigate for identification of a causative factor which is considerably important to improve muscle growth for production efficiency of food animals and a potential application to human health.

## Materials and Methods

### Ethics Statement

All animal procedures were approved by the Institutional Animal Care and Use Committee (IACUC) at The Ohio State University. All experiments were performed in accordance with the Prevention of Cruelty to Animals Act (1986).

### Animals, Muscle Samples, and Growth Performance

Fertile eggs from LW and RBC quail lines were obtained from The Ohio Agricultural Research and Development Center (OARDC) of the Ohio State University in Wooster (OH, USA). Fertile eggs were stored under normal storage condition (12°C with 70% relative humidity). Fertile eggs from the LW and RBC quail lines were incubated in a hatchery (Type 65Hs, Masallles, Spain). At 9, 11, 13, and 15 d of incubation, six eggs from each line (total 48 eggs) were randomly selected from the hatchery and weighed; and six embryos from each line were extracted and weighed. Embryo percentage was calculated as the ratio of embryo weight to egg weight. After hatching, quail from each line were reared in heated Petersime battery brooders with wire-mesh floors. Quail were maintained in battery cages grouped by each line, and were fed a commercial diet ad libitum in accordance with the National Research Council [28]. All animals were handled in compliance with the Institutional Animal Care and Use Committee policies and guidelines at The Ohio State University. At 0, 3, 7, 14, 21, 28, 42, and 75 d post-hatch, six quail from each line (total 96 quail) were randomly selected and weighed. Quail were individually euthanized by CO_2_ inhalation following standard procedures [29]. Pectoralis major muscles were exposed, and the entire right and left pectoralis major muscles were excised and weighed. The percentage of PMW was calculated as the ratio of the pectoralis major muscle weight to the body weight. Muscle samples were taken from the pectoralis major muscle including the superficial and deep regions, and immediately frozen in isopentane cooled by liquid nitrogen and stored at −80°C for the Western blot analysis and quantitative Real-Time PCR.

At 42 and 75 d post-hatch, the area of pectoralis major muscle was measured at the diagonal line of a point 1/2 in the pectoralis major muscle [5], [12]. A significant difference was observed in the lightness value (*L* ^*^) measured with a Minolta chromameter (CR-300, Minolta Camera Co., Japan) of the pectoralis major muscle between the superficial (away from the pectoralis minor muscle and bone) and deep (close to the pectoralis minor muscle and bone) regions at 42 and 75 d post-hatch [5]. Thus, area and percentage of each region in the pectoralis major muscle were measured separately. Muscle samples were also extracted from each of these regions, and then immediately frozen in isopentane cooled by liquid nitrogen and stored at −80°C for the histochemical analysis.

### Histochemical Analysis

#### Muscle Fiber Characteristics

Serial transverse muscle sections (10 µm) were obtained from each sample using a cryostat (CM 1510S, Leica, Germany) at −25°C, and then mounted onto glass slides. The myosin ATPase activity of the samples was detected following pre-incubation with acid (pH 4.6) or alkaline (pH 10.7) [31]. Muscle fibers were classified as type IIA and IIB fibers using the nomenclature system of Brooke and Kaiser [32]. Type I fibers were not found in the pectoralis major muscle of the LW and RBC quail lines [5]. All stained muscle samples at 42 and 75 d post-hatch were examined by an image analysis (Image-Pro Plus). At least 600 fibers were evaluated per sample. The average CSA of muscle fibers was determined as the ratio of total area measured divided by total number of muscle fibers, and CSA for type IIA and IIB fibers were also measured. Muscle fiber density (fiber numbers/mm^2^) was calculated from the mean number of fibers per mm^2^, as well as fiber density of each fiber type. Area percentage of each fiber type was calculated as the proportion of total CSA of each fiber type divided by total fiber area measured. Number percentage of each fiber type was calculated as the proportion of total number of each fiber type divided by total fiber numbers (TFN) measured. Total number of fibers was determined by multiplying muscle fiber density by the area of pectoralis major muscle. The total number of each region and total number of each fiber type were also measured.

#### Centered Nuclei Percentage

Cross dissected muscle tissues were fixed in 10% formalin, and embedded in paraffin. Ten-micrometer sections were obtained by cutting each region of the pectoralis major muscle, and subjected to hematoxylin and eosin (H & E) staining [30]. All stained muscle samples at 42 d post-hatch were examined by an image analysis system consisting of an optical microscope equipped with a CCD color camera (QImaging, Burnaby, BC, Canada) and a standard computer that controlled the image analysis system (Image-Pro Plus, Media Cybernetics, LP). At least 600 fibers were evaluated per sample. The percentage of centered nuclei was calculated as the proportion of centered nuclei divided by total nuclei under 200 X magnifications.

### Western Blot Analysis

Frozen muscle samples were homogenized in ice-cold 1 X lysis buffer (62.5 mM Tris, 5% SDS) with a Tissuemiser (Fisher Scientific, Pittsburgh, PA) and combined with 2 X Laemmli buffer (Bio-Rad Laboratories, Hercules, CA) containing 62.5 mM Tris, 1% SDS, 5% 2-mercaptoethanol, 12.5% glycerol, and 0.05% bromophenol blue. Proteins were separated by SDS-PAGE using a mini-Protein system (Bio-Rad Laboratory, Hercules, CA). Gels were stained with Coomassie brilliant blue and used as a control of protein loading. Proteins were wet-transferred to a 0.45 µm pore polyvinylidine fluoride membrane (Millipore, Billerica, MA). Membranes were blocked in 5% nonfat dry milk in tris-buffered saline-tween (TBST; 20 mM Tris, 150 mM NaCl, pH 7.4, plus 0.15% Tween 20) for 30 min at room temperature. Membranes were incubated overnight at 4°C in 5 different primary antibodies: 2E9 antibody (neonatal MHC isoform coded by the gene MYH8; 1∶1,000 dilution), AB8 antibody (which reacts with all adult fast MHC isoforms (IIA, IIX, and IIB) coded by the genes MYH2, MYH1, and MYH4, respectively; 1∶1,000 dilution), Pax7 antibody (1∶500 dilution; Developmental Studies Hybridoma Bank, Iowa City, IA), MyoD antibody (1∶1,000 dilution; Santa Cruz Biotechnology Inc., Santa Cruz, CA), and Myf-5 antibody (1∶500 dilution; Sigma-Aldrich Co., St Louis, MO). The MHC primary antibodies (2E9 and AB8) were a gift from Everett Bandman (University of California, Davis). Membranes were then washed in TBST for 1 h and incubated with horseradish peroxidase-conjugated secondary anti-mouse IgG (1∶5,000 dilution; Santa Cruz Biotechnology Inc.) or secondary anti-rabbit IgG (1∶5,000 dilution; Santa Cruz Biotechnology Inc.) in TBST for 3 h at room temperature. Membranes were washed again for 1 h in TBST followed by detection with ECL Plus (GE Healthcare, Piscataway, NJ). The membranes were exposed to Bio-Max X-ray film (GE Healthcare) for visualization of the neonatal MHC, adult MHC, Pax7, MyoD, and Myf-5 proteins. For the purpose of quantitative analysis, protein bands at specific time points (neonatal MHC: 3 and 75 d post-hatch; adult MHC: 28, 42, and 75 d post-hatch; Pax7: 7 and 14 d post-hatch; MyoD: 0, 3, and 7 d post-hatch; Myf-5: 3 and 7 d post-hatch) were examined by subtracting the background within the area measured for each band (Kodak 1-D Image Analysis Software, Eastman Kodak Company, Rochester, NY), and then compared, to the band intensities between the LW and RBC quail lines.

### Quantitative Real-Time PCR

At 0, 3, 7, 14, 28, 42, and 75 d post-hatch, muscle tissues from each line (6 samples for each time point) were used (total 84 samples). The total RNA were isolated according to the instructions of the manufacturer. A quantity of total RNA was measured using an ABI 7300 real-time PCR instrument (Applied Biosystems, Foster City, CA), and RNA quality was assessed from gel electrophoresis and normalized accordingly. Complementary DNA (cDNA) was synthesized using 500 ng of total RNA, according to procedures described in our previous report [33]. Relative mRNA expression levels of myogenin in the pectoralis major muscle between the two quail lines were assessed by quantitative PCR (qPCR). The sequences for forward and reverse primers were 5′-CTG CCC AAG GTG GAG ATC CT-3′ and 5′-GGG TTG GTG CCA AAC TCC AG-3′, respectively. Equal amounts of cDNA from each sample from the two quail lines were used as templates with the reaction system including AmpliTaq Gold polymerase (Applied Biosystems), GeneAmp 10×PCR Buffer (containing 100 mM Tris-HCl, pH 8.3), and 500 mM KCl. SYBR green was used to detect the amplification of the products. Duplicate reactions were performed (25 µL) on an ABI 7300 real-time PCR instrument (Applied Biosystems). The conditions of the qPCR reaction were 95°C for 10 min, 40 cycles of 94°C for 15 s, 60°C for 40 s, and 72°C for 30 s, with an additional 82°C for 33s. The comparative 2^-ΔCt^ method for relative quantification was used to calculate the relative gene expression. Ribosomal protein 13 (RPS13), which was tested to be the best housekeeping gene in quail [34], [35], was used to normalize the qRT-PCR calculation.

### Statistical Analysis

General linear model (GLM) procedure was performed for the association between quail lines using SAS software [36]. No significant difference was observed in any measurements between genders. Significant differences of values between the LW and RBC quail lines were detected by the probability difference (PDIFF), and mean values were separated at the level of 5%. The results for the lines are presented as least squares means together with standard errors.

## Results

### LW quail line showed a distinct growth pattern

**Figure 1:**
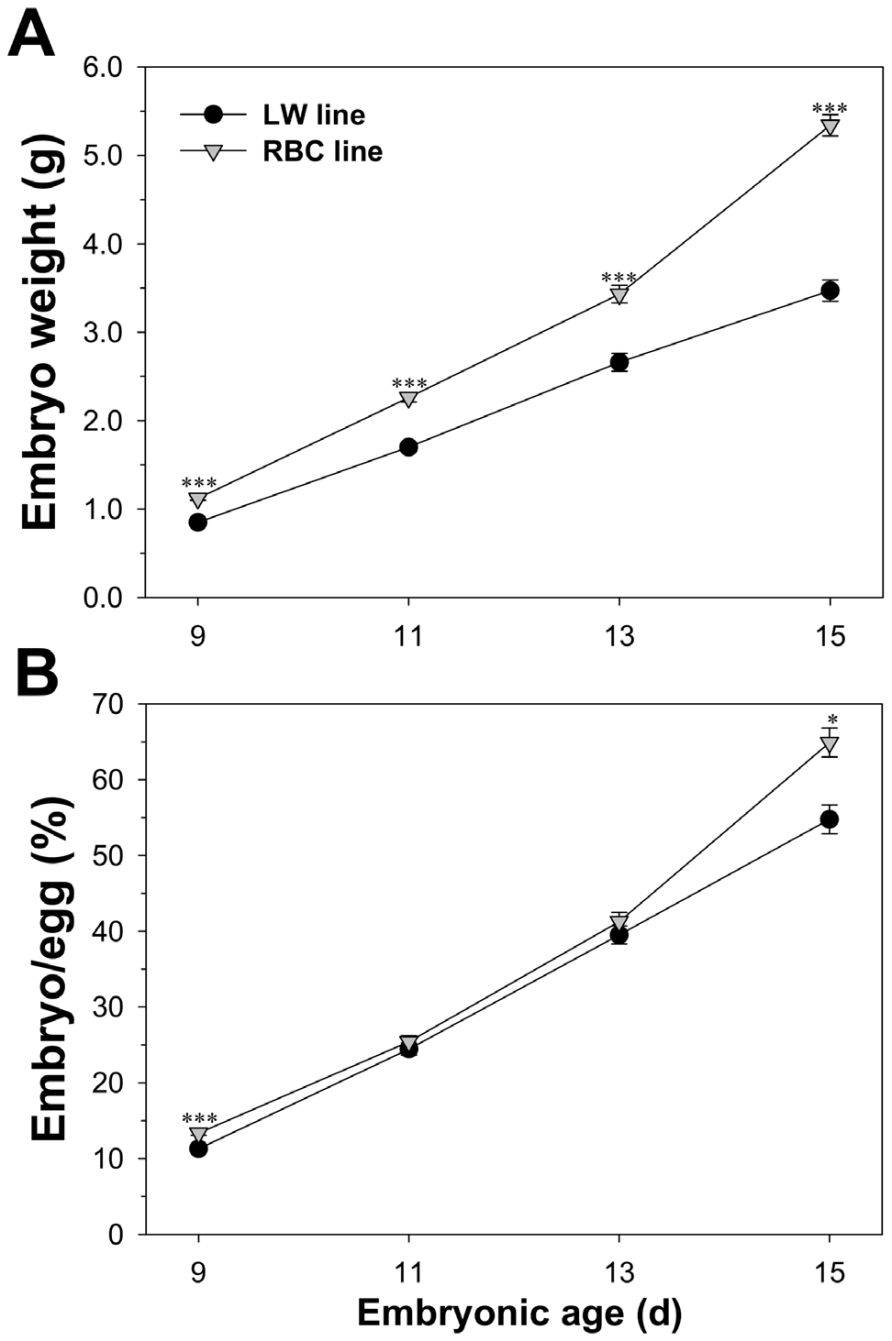
Comparison of embryo weight (A) and embryo percentage relative to egg weight (B) between the low weight (LW) and random bred control (RBC) quail lines. Bars indicate standard errors. Level of significance: * *P*<0.05; *** *P*<0.001.

Embryo weight and the ratio of embryo weight to egg weight of the two quail lines are presented in Figure 1. The LW quail line exhibited a lower embryo weight compared to the RBC quail line in all age groups (*P*<0.05). At 11 and 13 d of incubation, no significant differences were observed in the ratios of embryo to egg weights between the two quail lines (*P*>0.05). However, the LW quail line showed a smaller ratio compared to the RBC quail line at 9 (11.3 vs. 13.4%, *P*<0.001) and 15 (54.8 vs. 64.9%, *P*<0.001) d of incubation.

**Figure 2:**
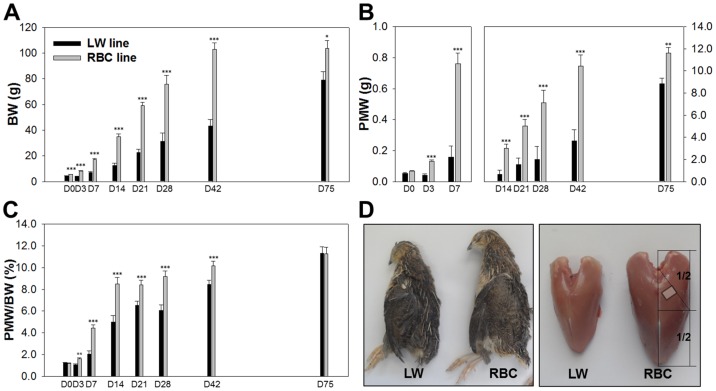
Comparison of body weight (BW, A), pectoralis major muscle weight (PMW, B), PMW percentage relative to BW (C), and schematic view of body and breast at 42 d post-hatch (D) between the low weight (LW) and random bred control (RBC) quail lines. The area of the pectoralis major muscle (D) was measured at the diagonal line of a point 1/2 in the pectoralis major muscle as reported by Scheuermann et al. [12]. Muscle samples were taken from the mid-point on the diagonal line for the histochemical analysis. Bars indicate standard errors. Level of significance: * *P*<0.05; ** *P*<0.01; *** *P*<0.001.

Body weight and pectoralis major muscle characteristics of the LW and RBC quail lines are presented in Figure 2 and Table 1. At 0 d post-hatch, body weight (4.4 vs. 5.6 g, *P*<0.001) and PMW (54 vs. 68 mg, *P*<0.001) of the RBC quail line were approximately 1.3 times heavier than those of the LW quail line. From 3 d to 42 d post-hatch, body weight and PMW were at least 2 times heavier in the RBC quail line compared to the LW quail line. A previous study reported by Choi et al. [2], [5], PMW were approximately 3.1 and 2.7 times greater for the HW quail line selected for a higher body weight compared to the RBC quail lines at 42 and 75 d post-hatch, respectively. In this study, body weight (79.7 and 104 g, *P*<0.05) and PMW (8.8 and 11.6 g, *P*<0.05) at 75 d post-hatch were approximately 1.3 times heavier for the RBC quail line compared to the LW quail line. The percentages of PMW relative to body weight were lower in the LW quail line compared to the RBC quail line at most of the time points except at 0 (1.2 vs. 1.2%, *P*>0.05) and 75 (11.3 vs. 11.3%, *P* = 0.965) d post-hatch.

**Table 1:** Comparison of pectoralis major muscle (PM) characteristics among the low weight (LW) and random bred control (RBC) quail lines at 42 and 75 d post-hatch.

	LW line	RBC line	SEM	*P-value*
*PM area (mm^2^) at 42 d post-hatch*				
Total	130	262	24.4	<0.001
Superficial region	65.5	84.3	20.1	0.185
Deep region	64.8	178	11.9	<0.001
*Percentage of each region in PM (%) at 42 d post-hatch*				
Superficial region	49.0	32.2	3.3	0.008
Deep region	51.0	67.8	3.3	0.008
*PM area (mm^2^) at 75 d post-hatch*				
Total	200	262	19.1	0.045
Superficial region	88.1	95.7	10.3	0.597
Deep region	112	166	12.3	0.013
*Percentage of each region in PM (%) at 75 d post-hatch*				
Superficial region	44.2	36.2	2.9	0.078
Deep region	55.8	63.8	2.9	0.078

The LW quail line had a smaller area of pectoralis major muscle at 42 (*P*<0.001) and 75 (*P* = 0.045) d post-hatch than the RBC quail line. Moreover, area of the deep region was smaller in the LW quail line compared to the RBC quail line at 42 (*P*<0.001) and 75 d post-hatch (*P* = 0.013). However, there was no significant difference in area of the superficial region between the different quail lines at 42 (*P* = 0.185) and 75 (*P* = 0.597) d post-hatch. Thus, the smaller size of the pectoralis major muscle in the LW quail line was accompanied by a smaller deep region area compared to the RBC quail line without a difference in the size of superficial region. The LW quail line exhibited a lower percentage of deep region at 42 d post-hatch (*P* = 0.008); whereas, no significant differences were found in the proportion of superficial (*P* = 0.078) and deep (*P* = 0.078) regions at 75 d post-hatch.

### Fiber hypoplasia affects muscle mass of LW quail line

**Figure 3:**
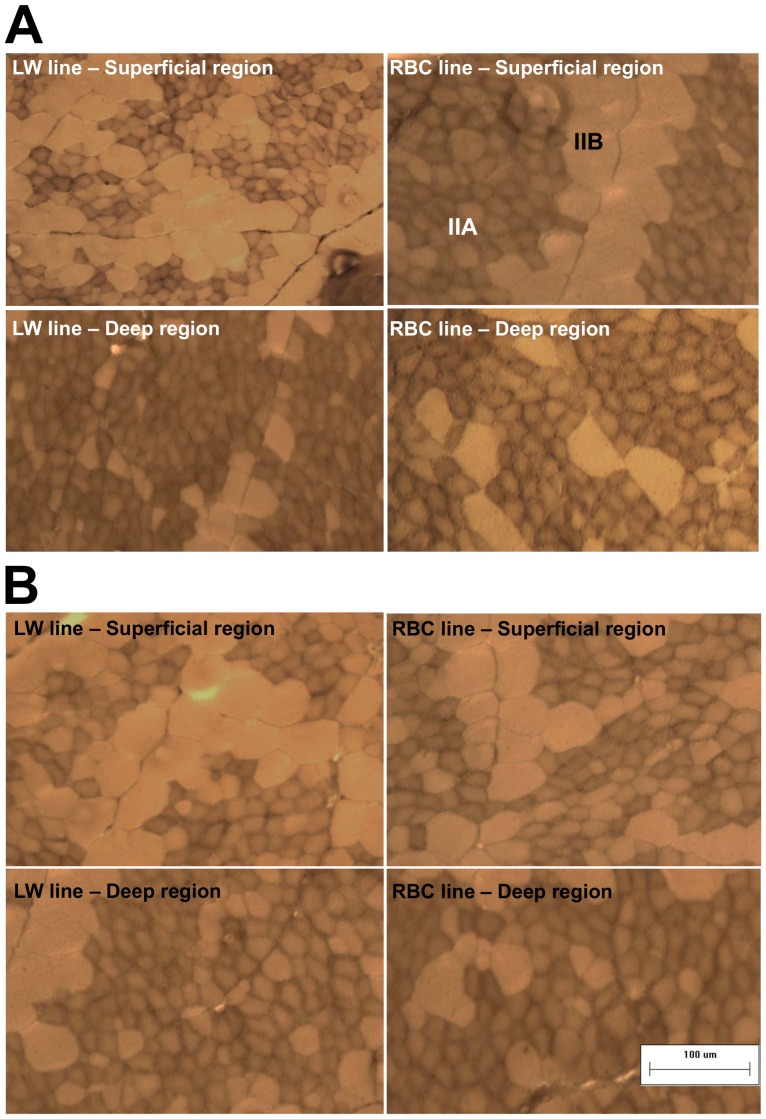
Muscle fiber cross-sections in the pectoralis major muscle between the low weight (LW) and random bred control (RBC) quail lines at 42 (A) and 75 (B) d post-hatch that were stained for myosin ATPase activity after acidic preincubation (pH 4.6). Bar indicates 100 µm. Abbreviations: IIA, fiber type IIA (fast-twitch and oxido-glycolytic); IIB, fiber type IIB (fast-twitch and glycolytic).

The superficial region had a higher percentage of larger type IIB fibers than the deep region in both quail lines (Table 2 and Figure 3). In the superficial region, no significant difference was observed in the area percentage of type IIA and IIB fibers between the LW and RBC quail lines (*P*>0.05), although the LW quail line had a lower number percentage of type IIA fibers than the RBC quail line at 42 d post-hatch (71.9 vs. 80.5%, *P* = 0.007). Fiber type composition in each region of the RBC or LW quail lines was similar between 42 and 75 d post-hatch (*P*>0.05). At 75 d post-hatch, the superficial region of the LW quail line showed a higher area percentage of type IIB fibers than that of the RBC quail line (59.3 vs. 48.5%, *P* = 0.008). Similar to 42 d post-hatch, the deep region of the LW quail line displayed similar area and number percentage of type IIA and IIB fibers compared to that of the RBC quail line at 75 d post-hatch (*P*>0.05).

**Table 2:** Muscle fiber type composition of the pectoralis major muscle on the different quail lines at 42 and 75 d post-hatch.

Muscle region	Superficial region		SEM	P-value	Deep region		SEM	P-value
Quail line	LW line	RBC line			LW line	RBC line		
*Muscle fiber area percentage (%) at 42 d post-hatch*								
Type IIA fiber	46.0	51.9	2.8	0.160	73.0	71.2	3.2	0.704
Type IIB fiber	54.0	48.1	2.8	0.160	27.0	28.8	3.2	0.704
*Muscle fiber number percentage (%) at 42 d post-hatch*								
Type IIA fiber	71.9^b^	80.5^a^	1.8	0.007	90.8	90.7	1.5	0.962
Type IIB fiber	28.1^a^	19.5^b^	1.8	0.007	9.2	9.3	1.5	0.962
*Muscle fiber area percentage (%) at 75 d post-hatch*								
Type IIA fiber	40.7^b^	51.5^a^	1.9	0.008	72.7	75.8	2.6	0.454
Type IIB fiber	59.3^a^	48.5^b^	1.9	0.008	27.3	24.2	2.6	0.454
*Muscle fiber number percentage (%) at 75 d post-hatch*								
Type IIA fiber	71.9^b^	80.5^a^	1.8	0.013	90.2	92.8	0.9	0.103
Type IIB fiber	28.1^a^	19.5^b^	1.8	0.013	9.8	7.2	0.9	0.103

a–b Different superscripts in the each muscle region represent significant differences (*P*<0.05).

Abbreviations: LW, low weight; RBC, random bred control.

**Figure 4:**
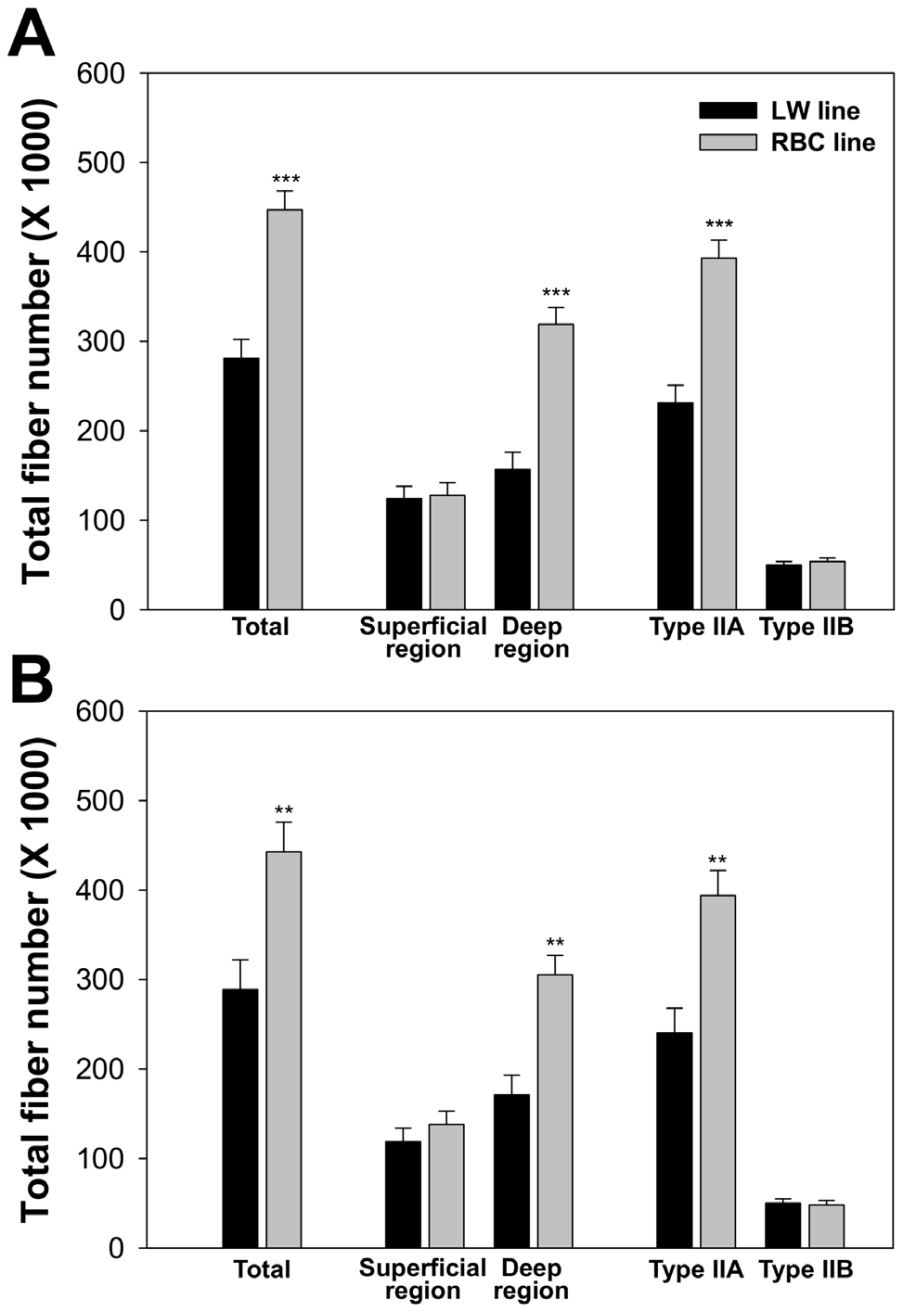
Comparison of total muscle fiber number in the pectoralis major muscle between the low weight (LW) and random bred control (RBC) quail lines at 42 (A) and 75 (B) d post-hatch. Bars indicate standard errors. Level of significance: ** *P*<0.01; *** *P*<0.001.

Within the LW or RBC quail lines, no significant difference was observed in TFN between 42 and 75 d post-hatch (*P* = 0.582 or 0.927, respectively) (Figure 4A and 4B). There were no significant differences in fiber number of the superficial region and type IIB fibers between the different quail lines at 42 and 75 d post-hatch (*P*>0.05). Whereas, fiber number of type IIA fibers was more than 1.6 times greater in the RBC quail line at 42 (*P*<0.001) and 75 (*P*<0.01) d post-hatch compared to the LW quail line. Moreover, fiber number of the deep region was about 50% less in the LW quail line (*P*<0.001), resulting in about 30% smaller TFN in the whole pectoralis major muscle at 42 (*P*<0.001) and 75 (*P* = 0.01) d post-hatch in the LW quail line compared to the RBC quail line. Although there was no significant difference in mean fiber CSA of the deep region between two lines of quail at 75 d post-hatch, as shown in Table 1 and 2, the 33% smaller deep region area of the LW quail line compared to the RBC quail line could be a main reason for the lesser number of total fibers in the whole pectoralis major muscle of the LW quail line.

### Delayed fiber hypertrophic potential observed in LW quail line

**Figure 5:**
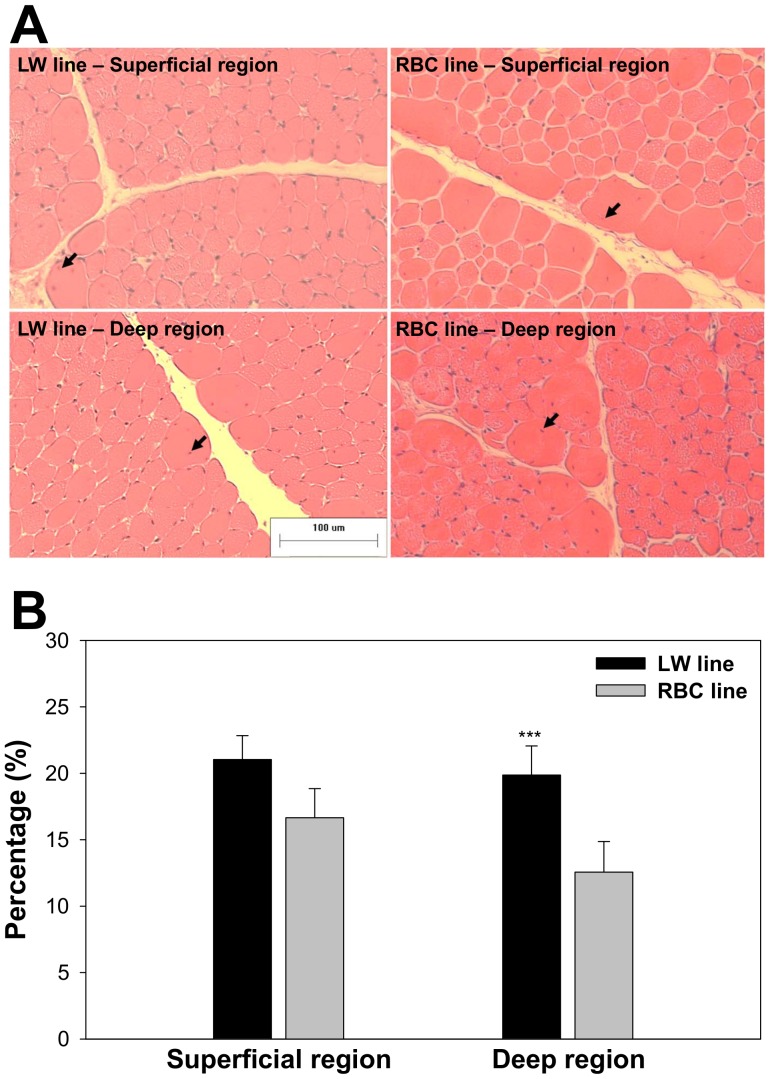
Muscle fiber cross-sections and centered nuclei in the pectoralis major muscle between the low weight (LW) and random bred control (RBC) quail lines at 42 d post-hatch that were stained for hematoxylin and eosin (A); Comparison of centered nuclei percentage relative to total counted nuclei between LW and RBC quail lines at 42 d post-hatch (B). Arrows indicate centered nuclei. Bars indicate standard errors. Level of significance: *** *P*<0.001.

Centered nuclei in the pectoralis major muscle of the different quail lines and different muscle regions at 42 d post-hatch are presented in Figure 5. The percentage of centered nuclei number relative to total nuclei number was significantly greater in the LW quail line compared to the RBC quail line (20.4 vs. 14.6%, *P*<0.01); whereas, no significant difference was observed in the superficial region between the LW and RBC quail lines at 42 d post-hatch (*P*>0.05).

The results for muscle fiber CSA and density of the pectoralis major muscle for the different quail lines at 42 and 75 d post-hatch are shown in Table 3 and Figure 3. The superficial and deep regions of the LW quail line exhibited smaller fiber CSA than those of the RBC quail line at 42 d post-hatch (*P*<0.05). From 42 to 75 d post-hatch, type IIA and IIB fiber CSA of the LW quail line were increased by 1.53 (313 to 478 µm^2^) and 1.55 (1107 to 1711 µm^2^) folds to the similar sizes of RBC quail line (*P*>0.05). The LW quail line had a higher fiber density than the RBC quail line at 42 d post-hatch (2369 vs. 1716 fibers/mm^2^, *P*<0.001). At 75 d post-hatch, due to increased CSA in the LW quail line after the 42 d, no significant difference was observed in fiber density between the LW and RBC quail lines (1471 vs. 1680 fibers/mm^2^, *P* = 0.077), even though the LW quail line had lower IIA (1216 vs. 1496 fibers/mm^2^, *P* = 0.008) and higher IIB (255 vs. 200 fibers/mm^2^, *P*<0.001) densities compared to the RBC quail line.

**Table 3:** Muscle fiber cross-sectional area (CSA) and density of the pectoralis major muscle on the different quail lines at 42 and 75 d post-hatch.

Muscle region	Superficial region		SEM	P-value	Deep region		SEM	P-value
Quail line	LW line	RBC line			LW line	RBC line		
*Muscle fiber CSA (µm^2^) at 42 d post-hatch*								
Mean	458^b^	661^a^	37.2	0.003	423^b^	561^a^	36.1	0.019
Type IIA fiber	289^b^	427^a^	23.8	0.002	337^b^	441^a^	33.0	0.032
Type IIB fiber	881^b^	1625^a^	21.7	<0.001	1325^b^	1745^a^	87.6	0.010
*Muscle fiber density (number/mm^2^) at 42 d post-hatch*								
Mean	2283^a^	1518^b^	141	0.005	2452^a^	1809^b^	164	0.027
Type IIA fiber	1648^a^	1225^b^	117	0.038	2235^a^	1665^b^	172	0.046
Type IIB fiber	636^a^	293^b^	41.6	<0.001	217	165	31.8	0.258
*Muscle fiber CSA (µm^2^) at 75 d post-hatch*								
Mean	740	719	31.7	0.677	650	554	35.3	0.112
Type IIA fiber	418	459	21.5	0.253	525	454	38.5	0.256
Type IIB fiber	1578	1799	90.7	0.111	1818	1847	114	0.856
*Muscle fiber density (number/mm^2^) at 75 d post-hatch*								
Mean	1357	1408	65.4	0.583	1563	1818	99.6	0.098
Type IIA fiber	974	1138	57.6	0.100	1408^b^	1685^a^	84.7	0.045
Type IIB fiber	383^a^	270^b^	24.7	0.018	155	133	22.0	0.475

a–b Different superscripts in the each muscle region represent significant differences (*P*<0.05).

Abbreviations: LW, low weight; RBC, random bred control.

### Expressions of MHC isoforms are delayed in LW quail line, and myogenic markers are differentially expressed in LW and RBC quail lines

**Figure 6:**
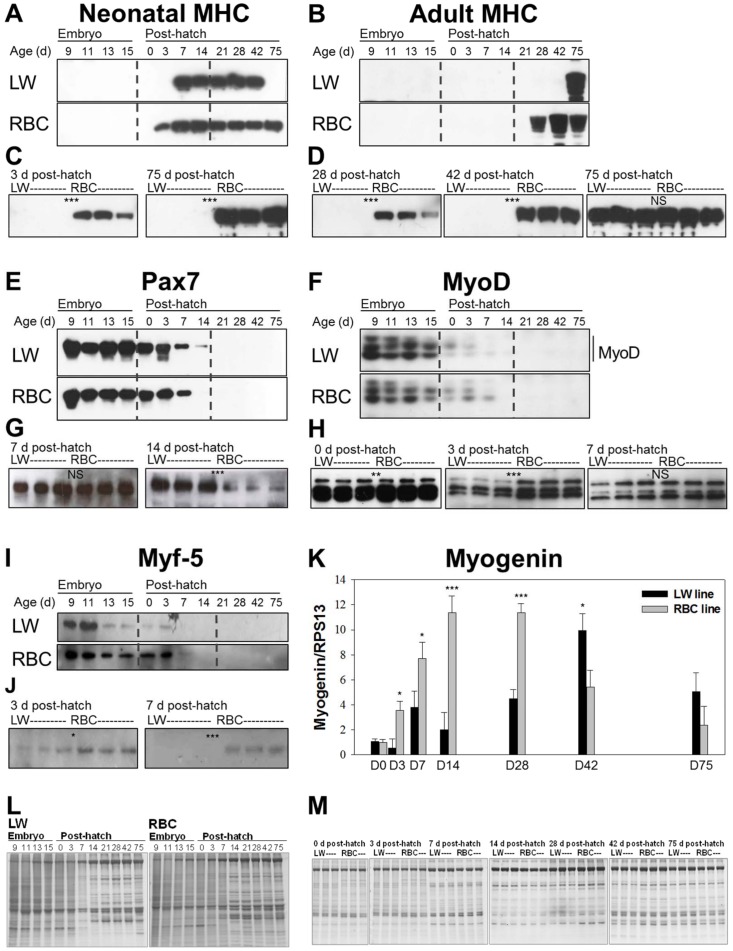
Expression of the different myosin heavy chain (MHC) isoforms and myogenic factors in the low weight (LW) and random bred control (RBC) quail lines. Time point comparison of the neonatal MHC isoform using 2E9 monoclonal antibody (A), adult MHC isoform using AB8 monoclonal antibody (B), Pax7 (E), MyoD (F), and Myf-5 (I) between the LW and RBC quail lines (1 quail per each time point). Quail line comparison of neonatal MHC isoforms at 3 and 75 d post-hatch (C), adult MHC isoform at 28, 42, and 75 d post-hatch (D), Pax7 at 7 and 14 d post-hatch (G), MyoD at 0, 3, and 7 d post-hatch (H), and Myf-5 at 3 and 7 d post-hatch (J) (3 quail per each time point and each quail line). Expressions of myogenin mRNA in the two quail lines at 0, 3, 7, 14, 28, 42, and 75 d post-hatch (K). Total RNA was isolated from the pectoralis major muscle. Expressions of myogenin were measured by quantitative real-time PCR with avian ribosomal protein 13 (RPS13) as a control for normalization. Protein staining of the different quail lines at each time point (L; 1 quail per each time point) and at 0, 3, 7, 14, 28, 42, and 75 d post-hatch (M; 3 quail per each time point and each quail line). Gels used as a control of protein loading. Bars indicate standard errors. Level of significance: NS, not significant; * *P*<0.05; ** *P*<0.01; *** *P*<0.001.

Temporal expressions of MHC isoforms and myogenic markers are assessed by western blot analysis, and graphically presented in Figure 6. Differences in MHC isoforms and myogenic markers expression between the different quail lines across all experimental time points were investigated first (n = 1). This initial view of temporal expression patterns allow us to focus on specific time points when there were obvious differences, to further compare three individuals per group. The 2E9 primary antibody, which is specific for the neonatal MHC isoform, reacted with MHC in the LW quail line from 7 to 42 d post-hatch (Figure 6A); whereas, its expression had a much wider range of time period from 3 to 75 d post-hatch in the RBC quail line (Figure 6C). This isoform was then abolished in the LW quail line at 75 d post-hatch, whereas in the RBC quail line it persisted until 75 d post-hatch. Moreover, the neonatal MHC isoform was only expressed in the RBC quail line at 3 d post-hatch. The AB8 antibody detected the adult MHC isoform from 28 through 75 d post-hatch in the RBC quail line; whereas, the adult MHC isoform was found only at 75 d post-hatch, which was the predominant isoform expressed in the LW quail line (Figure 6B). However, there was no significant difference in the expression level of the adult MHC isoform between the different quail lines at 75 d post-hatch (Figure 6D). Thus, expressions of MHC isoforms were delayed in the LW quail line compared to the RBC quail line.

Comparison of individual expression of Pax7 showed that expression continued at 14 d post-hatch in the LW quail line; whereas, expression was not detected at 14 d post-hatch in the RBC quail line (Figure 6E). Comparison of the quail lines at 7 d post-hatch exhibited no significant difference in the expression level of Pax7. At 14 d post-hatch, although over-exposure of the x-ray film from 1 to 5 min could detect Pax7 band in the RBC quail line (Figure 6G), the expression level of this protein was significantly greater in the LW quail line compared to the RBC quail line at 14 d post-hatch (*P*<0.001).

Comparison of individual expression of MyoD demonstrated that expression continued at 7 d post-hatch in the LW and RBC quail lines (Figure 6F). However, significantly lower expression level showed at 0 (*P*<0.01) and 3 (*P*<0.001) d post-hatch in the LW quail line compared to the RBC quail line (Figure 6H). Comparison of individual expression of Myf-5 exhibited that expression continued at 7 d post-hatch in the RBC quail line, while expression was not detected at 7 d post-hatch in the LW line (Figure 6I). Similar to MyoD expression, the LW quail line showed a lower expression level of Myf-5 at 3 d post-hatch (Figure 6J). At 7 d post-hatch, a higher band intensity of Myf-5 was observed in the RBC quail line compared to the LW quail line (*P*<0.001); however there was no significant difference in expression level of MyoD between the quail lines. In quantitative real-time PCR (Figure 6K), the LW quail line showed lower myogenin expression levels compared to the RBC quail line during 3 to 28 d post-hatch (*P*<0.05). Thereafter, the LW quail line exhibited higher expression levels at 42 d post-hatch (9.9 vs. 5.4, *P*<0.05). However, no significant difference was observed at 75 d post-hatch between the two quail lines, although the LW quail line exhibited a higher expression level compared to the RBC quail line (5.1 vs. 2.4, *P*>0.05). Thus, myogenic regulatory factors were differentially expressed in the LW and RBC quail lines.

## Discussion

The LW quail line has a distinct growth pattern compared to the RBC quail line. The LW quail line exhibited smaller embryo sizes than the RBC quail line in all embryonic age groups. These differences in embryo growth between the two quail lines could be explained by a study conducted by Lilja and Olsson [37], who reported that the difference in embryo growth has been presumed to be due to differences in egg size, and corresponding nutrient availability. In this study, the LW line had lower egg weights compared to the RBC line (6.9 vs. 8.5 g, *P*<0.001), which might affect the growth rates of embryos in the two quail lines. The RBC line harboring a greater birth weight had approximately three times greater PMW than the LW line harboring a lower birth weight at 42 d post-hatch due to the results of selection (*P*<0.001). Thus, the growth pattern of the LW quail somewhat supports the previous finding that low birth weights are generally accompanied with lower potential growth rates, and associated with lower fiber hyperplastic potential [38], [39]. In the current study, the LW quail are an unique bird model with lower weight and smaller area of pectoralis major muscle at 42 d post-hatch that were mainly associated with muscle fiber hypotrophy and hypoplasia, especially a lower fiber number of deep region (*P*<0.001) and type IIA fiber number (*P*<0.001), compared to the RBC quail line.

However, as the LW quail has been selected from the RBC quail population on the basis of their lower body weight at 4 weeks of age, the growth of the LW line was much slower than the RBC line up to 4 weeks of age. Thereafter, differences in body weight (2.4 to 1.3 times) and PMW (3.6 to 1.3 times) between the RBC and LW quail lines diminished with increasing age from 28 to 75 d post-hatch. This is due mainly to the fact that the LW line continued to grow from 42 to 75 d post-hatch unlike the RBC line. PMW of the LW quail line at 75 d post-hatch was more than 2 times heavier than that of the LW quail line at 42 d post-hatch (8.8 vs. 3.7 g, *P*<0.001); whereas, TFN (*P*<0.001) was still smaller in the LW line compared to the RBC line at 75 d post-hatch. This later growth of the LW quail line is accompanied by a later increase in muscle fiber CSA from 42 to 75 d post-hatch (440 vs. 698 µm^2^, *P*<0.001) and a higher centered nuclei percentage in the deep region of the pectoralis major muscle at 42 d post-hatch (19.9 vs. 12.6%, *P*<0.05) suggesting delayed muscle maturation [40], [41]. Therefore, the most important time window affecting ultimate muscle mass is different between the LW and RBC quail lines. These delayed muscle maturation and fiber hypertrophic potential in the LW line may be associated with a late catch-up growth (acceleration of growth rate). Catch-up growth has been characterized by rapid growth after a temporary period of growth retardation occurring in humans and animals [42]. However, its accurate mechanisms are not clear, and the LW quail line can be a good model for understanding the phenomenon of late catch-up growth.

An outstanding increase of growth performance can be achieved through selection, as well as several concomitant alterations in muscle fiber characteristics, especially muscle fiber type composition [43]. Generally, animals selected by their muscle growth rate had muscles with a higher percentage of larger type IIB fiber and a lower percentage of smaller type I fiber [12], [44], [45]. In the current study, the LW quail line selected for a lower body weight did not differ considerably in fiber type composition compared to the RBC quail line at 42 and 75 d post-hatch. Thus, fiber type composition was found to have only a limited effect on body and muscle weights between the different quail lines.

During muscle development, several distinct MHC isoforms progressively appeared within the same muscle [16], [18]. The nomenclature of MHC isoforms (embryonic, neonatal, and adult isoforms) indicates their expression periods during muscle development, although embryonic and neonatal MHC isoforms actually persisted in fetal and adult muscles, respectively [46]. During postnatal development, embryonic and neonatal MHC isoforms are sequentially abolished at various time periods, and MHC transitions occur with age according to changes in muscle characteristics toward metabolic maturation for gaining contractile ability [18]. In mice, the neonatal MHC isoform in the plantaris muscle (fast-twitch muscle) was rapidly replaced by the adult MHC isoforms after birth; whereas, this isoform in the soleus muscle (slow-twitch muscle) persisted until 21 d after birth [17]. Lee et al. [21] reported that the adult MHC isoform was first detected after 11 d post-hatch in the pectoralis major muscle of broiler chickens, whereas the replacement of neonatal by adult MHC isoform was first shown at 27 d post-hatch in the pectoralis major muscle of the Leghorn chickens. In this study, clear differences were observed in the MHC isoform transition between the different quail lines. The neonatal MHC isoform was detected earlier in the RBC line compared to the LW line. In addition, the expression of the adult MHC isoform began at 28 d post-hatch in the RBC line, whereas expression was only detected at 75 d post-hatch in the LW line. This neonatal to adult MHC transition that occurs during development is associated with the time of skeletal muscle maturation [6]. In the current study, delayed maturation of postnatal muscle in the LW quail line may allow the later hypertrophic growth of muscle that has occurred much earlier in the RBC quail line.

The adult MHC isoform in the pectoralis major muscle is a predominant isoform of normal chickens during muscle maturation; whereas, the abundance of the neonatal isoform and lack of the adult isoform were observed in the adult dystrophic chickens [6]. Thus, muscular dystrophy, which is a developmental disorder, is associated with the MHC composition or interference of the transitions of MHC isoforms during muscle maturation [6]. In the current study, both the quail lines showed normal physical activities including walking and flying abilities as assessed at 75 d post-hatch. Thus, unlike muscle dystrophic chickens with an absence of the adult MHC isoform, the LW quail line with the delay in transition to the adult MHC isoform and developmental process of skeletal muscle could serve as a unique model.

It is generally accepted that muscle fiber number is fixed before birth in most terrestrial vertebrates [43], [47]. In large mammals, such as cattle, sheep, and human, the major events of contractile and metabolic differentiation occur during the last period of gestation, and are fully achieved soon after birth [47]. In cattle, embryonic and neonatal MHC isoforms are completely supplanted by adult MHC isoforms at the end of gestation [48]. However, in rodents and birds that are prematurely born in terms of physical activity, maturation of muscle fiber occurs during the first postnatal month when the neonatal MHC isoform is disappearing and the adult MHC begins to be dominantly expressed [21], [49], [50]. Interestingly, the LW line (42 to 75 d post-hatch) have a lot of delay at the beginning the transition of neonatal to adult MHC isoforms compared to the RBC line (28 d post-hatch), which is associated with different times of gaining flying abilities between the two quail lines. In addition, the initial time of the neonatal MHC expression and expression of early myogenic markers in the quail breast muscle after hatch, which generally occurs in the fetal muscle of the large mammals, further suggests that development of quail muscle within one week after hatch could be equivalent to the stages of muscle development with the late fetal ages of large mammals. In this regard, these differences in the kinetics of muscle fiber development between species are associated with their maturity at birth, and delayed muscle growth in less mature species is accompanied by less physical ability at birth compared to large mammals [47]. In fact, most of the small volant species including quail have achieved the requirements for high frequency operation and resistance to fatigue associated with hovering and flapping flight during the first few weeks after post-hatch due to their immature muscle fiber structure and feather formation, while most of the large vertebrates have achieved the physical ability to perform the movement shortly after birth. Considering the late development of muscle maturation in the quail, the time window for muscle fiber formation, especially in breast muscle, may be extended to the early post-hatch period in quail. This is further supported by the finding that muscle fiber hyperplasia in animals is largely completed before or after birth or hatch depending on times of maturation of various muscles [47], [51], [52]. The muscles used right after birth or hatch tend to mature sooner, and their fiber numbers are determined earlier than the other muscles that require being fully functional at a much later time. For example, in the rectus femoris muscle of mice, which is necessary for lifting the leg, the fiber number is fixed around the time of birth [53]. Whereas, the fiber number of the medial pterygoid, a quadrilateral muscle for mastication, increased by two-fold from birth to 6 weeks in the rats, perhaps due to the required gaining of strength and mass of the pterygoid during the transition from sucking milk to eating solid chow diets [51]. Considering the immature breast muscle of the quail chick at hatch, it is possible that the fiber number may be increased during the early post-hatch. It will be interesting to evaluate an increase in fiber number, although our trial for measuring fiber number at early post-hatch was hampered by limitations of counting the total number in a cross section, such as fiber branching, nascent short fibers being longitudinally arranged in series, and difficulty in decision on exclusion or inclusion of very small-fiber like shapes.

Pax7 is strictly expressed in myogenic and satellite cells and induces myoblast proliferation [23]. Pax7 is down-regulated during differentiation [23]. After the differentiation period, high levels of Pax7 could inhibit myogenesis and induce cell cycle exit even in growth conditions to maintain satellite cell population [54]. In this study, Pax7 expression persisted longer in the LW quail line until 14 d post-hatch compared to the RBC quail line. These results suggest delayed satellite cell proliferation and myogenic differentiation in the LW quail line, which is further evidenced by the delayed expressions of neonatal and adult MHC isoforms in the LW line compared to the RBC line.

Rudnicki et al. [55] reported that MyoD and Myf-5 play crucial roles in the differentiation of muscle precursor cells to myogenic cells. The essential roles of MyoD and Myf-5 in myogenesis were further evidenced by the finding that double mutant mice [*MyoD(-/-);Myf-5(-/-)*] are unable to form muscle fibers, a result of absent myoblasts [26]. Prolonged expression of Pax7, which is associated with delayed muscle growth in the LW quail, led to further investigate expression of myogenic factors involved in differentiation, the next step after Pax7 in the general processes of myogenesis. In this study, the LW quail line showed lower expression levels of MyoD (0 and 3 d) and Myf-5 (3 and 7 d) at the early post-hatch period compared to the RBC quail line. In fact, amounts and time of expression of these proteins could be major factors affecting the population of differentiated myocytes that contribute to formation of muscle fibers [26]. Therefore, higher expression levels of primary MRFs at the early post-hatch period suggest that the myogenic differentiation was earlier and stronger in the RBC quail line, while lower expression levels of primary MRFs in the LW quail line could suppress sequential processes of myogenesis, consequently resulting in a reduction of total fiber number.

In support of this, expression levels of myogenin were significantly lower in the LW quail line during early post-hatch periods compared to the RBC quail line (*P*<0.05). Myogenin is a secondary MRF acting downstream of primary MRFs, MyoD and Myf-5, and the absence of myogenin prevented skeletal myoblasts from contributing to postnatal muscle hyperplastic and hypertrophic growth [56]. Knapp et al. [57] reported that low expression level of myogenin in postnatal period leads to the reduced body weight, but normal skeletal muscle in mice. Taken together, up-regulation of Pax7 and down-regulation of MyoD, Myf-5 and myogenin suggest that the LW quail likely have impairment in the differentiation stage of myogenesis, which consequently leads to hypoplastic muscle growth in the LW line at the early post-hatch period. Contrarily, higher levels of myogenin expression at 42 d post-hatch were associated with a higher hypertrophic potential of the LW quail line, which support the delay in muscle growth with the remaining lower number of muscle fibers. These data further support that muscle fiber numbers are determined within the time window from embryonic to the early post-hatch period in quail.

## Conclusion

The results of this study are the first comparison of physiological, morphological, and developmental parameters between the LW and RBC quail lines during muscle development. Overall, the results indicate that muscle fiber hypoplasia has more impact on body and muscle weights of the LW quail line selected for a lower body weight at 4 weeks of age compared to muscle fiber hypotrophy. However, a late catch-up growth via delayed muscle fiber hypertrophy was observed in the LW quail line between 42 and 75 d post-hatch, which associated with a higher percentage of centered nuclei and a higher expression level of myogenin at 42 d post-hatch. Muscle fiber hypoplasia and delayed growth pattern in the LW quail line may be influenced by the delayed transition of neonatal to adult MHC isoform and expression time and expression levels of Pax7 and MRFs. The most important time window affecting muscle fiber hypoplasia in LW quail line was between 0 to 3 d post-hatch. At this period, the LW quail line showed decreased expression levels of myogenin (0.5 vs. 3.6 folds) and impaired pectoralis major muscle growth (0.9 vs. 1.4 folds) compared to the RBC quail line. The results from the current study provide a useful avian model for understanding the mechanisms underlying decrease in muscle mass due to impaired and delayed muscle growth during development and increased hypertrophic potential during maturation. To practically apply this knowledge for the improvement of muscle mass in food animals and human health, more information must be gathered on causative events during development and identification of genes responsible for muscle fiber hypoplasia and delayed fiber hypertrophy in the LW quail.
